# Percepção de pacientes sobre a comunicação médica e suas necessidades durante internação na unidade de cuidados intensivos

**DOI:** 10.5935/0103-507X.20210050

**Published:** 2021

**Authors:** Marlon Corrêa, Flávia Del Castanhel, Suely Grosseman

**Affiliations:** 1 Curso de Medicina, Centro de Ciências da Saúde, Universidade Federal de Santa Catarina - Florianópolis (SC), Brasil.; 2 Programa de Pós-Graduação em Ciências Médicas, Universidade Federal de Santa Catarina - Florianópolis (SC), Brasil.

**Keywords:** Cuidados críticos, Comunicação, Preferência do paciente, Satisfação do paciente, Conforto do paciente, Relações médico-paciente

## Abstract

**Objetivo:**

Conhecer a percepção de pacientes sobre a comunicação médica, bem como suas necessidades durante internação na unidade de cuidados intensivos.

**Métodos:**

Estudo transversal descritivo e qualitativo exploratório, com 103 pacientes internados ou com alta recente da unidade de cuidados intensivos de quatro hospitais da Grande Florianópolis (SC). Foram estudadas variáveis sociodemográficas e clínicas dos pacientes, sua nota para qualidade da comunicação médica pelo *Quality of Communication Questionnaire*, seus comentários espontâneos com reflexões ou justificativas para as notas dadas e suas respostas sobre como se sentiam e que ajuda complementar gostariam de receber. Os dados quantitativos foram analisados com estatística descritiva e analítica e os qualitativos com análise de conteúdo temática.

**Resultados:**

A média do *Quality of Communication Questionnaire* foi 5,1 (desvio-padrão - DP = 1,3), sendo 8,6 (DP = 1,3) na subescala de comunicação geral e 2,1 (DP =1,8) na de terminalidade de vida. A linguagem médica teve compreensão variável. Alguns médicos pareciam “apressados”, segundo alguns pacientes. Outros pacientes gostariam de informações mais frequentes e detalhadas e/ou serem respeitados e levados “mais a sério” quando referiam sentir dor. Ansiedade, tristeza e/ou medo estavam entre os sentimentos referidos. Outras necessidades abrangeram silêncio, mais tempo para visitas, presença de acompanhante, atenção psicológica e de serviço social, banheiro que pudessem usar e melhor qualidade da comida na unidade de cuidados intensivos.

**Conclusão:**

A qualidade da comunicação médica com os pacientes é boa e poderia melhorar com maior disponibilidade de tempo do médico e da equipe para ela. Outras necessidades sentidas incluem respeito, alívio da dor e adaptações na dinâmica e no ambiente da unidade de cuidados intensivos.

## INTRODUCTION

Hospitalization in an intensive care unit (ICU) can be a stressful experience for patients,^(1-3)^ whose memory of it may affect their physical and psychological recovery for a considerable time after discharge.^(4-9)^

The ICU environment and care dynamics,^(1-5,7-19)^ health conditions, and the communication with physicians and the healthcare team are some factors that influence patient experience.^(5,7-19)^

Communication is the process by which human beings interact and share knowledge, thoughts, and feelings, with words (verbal) and without words (nonverbal), for example, with their look and gestures. Quality communication is essential for physicians to deliver care, as it enables the rapport-building and maintenance with patients, a more accurate diagnosis, and greater treatment adherence. Communication should focus on the relationships established at each moment of the clinical encounter between patients and people involved in their care and should include several elements, such as friendliness; attention; respect; attentive or active listening to patient problems, perspectives, needs, and expectations; recognition of and empathic responses to patient emotions; clear language when sharing information; and agreeing on the therapeutic plan.^(20)^

While effective communication tends to be therapeutic and to create security and reduce the trauma of hospitalization,^(3,5,9,11-13,16,17,19)^ its inadequacy can cause anxiety and stress.^(17)^

Several things can lead health professionals who work in the ICU to not value communication with patients as one of their main focus, sometimes without realizing it, which may result in communication failure.^(2,5,7-9,14)^

Evaluating the quality of medical communication with ICU patients and understanding their needs are essential in order to promote the quality of their care.^(1,3,9,13,17)^

The Quality of Communication Questionnaire (QoC)^(21,22)^ was developed by Curtis et al. in patients with HIV, cancer, or oxygen-dependent chronic obstructive pulmonary disease to evaluate the quality of medical communication with end-of-life patients.^(22,23)^ Because it contains a general communication subscale, the QoC has also been used in patients who are not in the end-of-life.

With the permission of the authors, the QoC has been translated and adapted for Brazilian patients admitted to the ICU,^(24)^ who were not necessarily in the end-of-life. Later, for its validation (in progress), the Brazilian version^(24)^ was applied to inpatients or patients recently discharged from the ICU and to patients who were in the end of their lives. When answering the QoC, patients spontaneously made reflections or comments to justify the score assigned to each item, similar to what was found by Russell in a study in Australia.^(5)^

Considering the importance of valuing the comments of patients when assigning scores on the QoC, and their testimonies about their needs during their ICU stay, the objective of this study was to investigate patients’ perception about medical communication, as well as their needs, during their ICU stay.

## METHODS

This cross-sectional, descriptive and qualitative exploratory study was part of a larger project for validation of QoC that is in progress and was approved by the ethics committee under number 77721917.8.3003.5355.

### Population and study site

The present study included only patients admitted to the ICU or who were in the ward after recent ICU discharge in four public hospitals in Greater Florianópolis (Santa Catarina, Brazil). The inclusion criteria were being 18 years of age or older, being awake and lucid, having an adequate level of consciousness to be interviewed, having no problems that could interfere with communication, and being hospitalized for more than 24 hours. The sample was selected by convenience.

The selection was by convenience, with invitation to participate in the study to patients who were hospitalized at the time of one of the researcher’s visits, which were performed daily on weekdays.

### Data collection

The data were collected in a face-to-face interview with each patient by a previously trained researcher. After the interviewer explained the study objectives, the data collection form, and all ethical principles, eligible patients were invited to participate. Those who agreed received two copies of the informed consent form for reading and signing, and kept one of them.

Data were collected using a structured questionnaire containing sociodemographic variables (age, sex, educational level and marital status), cause of ICU admission, the QoC and two open questions: “How are you feeling?” and “What type of complementary help would you like to receive?”.

The QoC consists of 13 items. The first six comprise subscale 1 and are related to general communication, and the last seven comprise subscale 2 and are related to end-of-life communication.^(22)^ The items are answered on a scale from zero (“worst imagined”) to ten points (“best imagined”). There are two alternative options: “Did not do”, scored as zero, and “do not know”, scored with the median of the scores assigned by the patient in the other items. The QoC score is calculated by the mean of its 13 items, the score of subscale 1 by the mean of items 1 to 6 and that of subscale 2 by the mean of items 7 to 13.^(22)^

After reading the QoC statement, the interviewer read each item. When necessary, the statement was read again, emphasizing that the score was for medical communication. The scores and spontaneous comments by the interviewees justifying them, or reflections on other aspects of communication, including with the healthcare team, as well as the answers to the open questions, were recorded in writing.

### Data analysis

The software used was the Statistical Package for the Social Sciences (SPSS), version 26.0. For data analysis, descriptive statistics, and, in order to analyze differences between two groups, Student’s t-test (t) for continuous variables and chi-square test (χ^2^) for categorical variables were used and two-way between-group analysis of variance (F) was used to analyze the impact of educational level, sex and marital status on QoC scores. The null hypothesis was rejected when p < 0.05. The degrees of freedom (df) are presented in parentheses immediately after the indication of the test performed.

A content analysis was performed for the qualitative data, consisting of an initial floating reading to familiarize with the data, followed in sequence by identification of meaning units, context units and categories.^(25)^

## RESULTS

### Patient profile

Among the 103 patients who participated in this study, 76 were in the ICU (73.8%) and 27 were in the ward after recent discharge from the ICU (26.2%) (χ^2^ (1) = 23.31, p = 0.000); 48 were women (46.6%) and 55 were men (53.4%) (χ^2^ (1) = 0.48, p = 0.49). Thirteen patients were single (12.6%), 80 were married or in a stable relationship (77.7%), eight were widowed (7.8%), and two were divorced (1.9%) (χ^2^ (3) = 154.75, p = 0.000). A total of 49 had an incomplete (47.6%) and 22 had a complete basic education (21.4%), six had an incomplete (5.8%) and 18 had a complete secondary education (17.5%), four had an incomplete (3.9%) and four had a complete higher education (3.9%) (χ^2^ (5) = 87.89, p = 0.000). The mean age was 51.8 years, with standard deviation (SD) of 14.7, with no difference by sex (t (101) = 1.59, p = 0.11).

Table 1 shows the causes of ICU admission.

**Table 1:** Cause of admission to the intensive care unit

Origin and cause of ICU admission	n (%)
Cardiovascular (n = 31)	
Myocardial revascularization/saphenous bypass	7 (6.8)
Catheterization	4 (3.9)
Pulmonary thromboembolism	3 (2.9)
Angioplasty	3 (2.9)
Heart failure	2 (1.9)
Acute myocardial infarction	2 (1.9)
Thrombosis	2 (1.9)
Aortic valve implant or replacement	2 (1.9)
Other*	6 (5.8)
Respiratory or noncardiovascular thoracic (n = 25)	
Chronic obstructive pulmonary disease/pulmonary emphysema	5 (4.8)
Pneumonia	7 (6.8)
Tuberculosis	3 (2.9)
Respiratory failure	4 (3.9)
Lung biopsy or partial to total resection	6 (5.8)
Gastrointestinal/abdominal (n = 13)	
Upper gastrointestinal bleeding	2 (1.9)
Liver cirrhosis	2 (1.9)
Partial gastrectomy	3 (2.9)
Partial enterectomy	3 (2.9)
Other†	3 (2.9)
Neurological (n = 11)	
Stroke	5 (4.8)
Traumatic brain injury	2 (1.9)
Craniotomy	2 (1.9)
Other‡	2 (1.9)
Renal (n = 6)	
Chronic renal failure	2 (1.9)
Pyelonephritis	2 (1.9)
Other§	2 (1.9)
Other origin (n = 17)	
Sepsis	6 (5.8)
Multiple trauma	2 (1.9)
Exogenous intoxication	2 (1.9)
Leptospirosis	2 (1.9)
Other¶	5 (4.8);
Total	103 (99.2)||

* Unstable angina, intracardiac tumor resection, cardioverter-defibrillator implantation, cardiac arrhythmia, infective endocarditis and aortic endoprosthesis (1 each); †liver transplantation, hepatectomy, laparotomy (1 each); ‡acute seizures and spinal cord trauma (1 each); §acute renal failure, nephrectomy (1 each); ¶Guillain-Barré syndrome, HELLP syndrome (hemolysis, high levels of liver enzymes, and low platelet count), prosthesis implant (unspecified), cervical fracture and pelvis and femur fixation (1 each); ||the total percentage is not equal to 100% due to rounding to one decimal place. Two patients had chronic renal failure, and 5 had oxygen-dependent chronic obstructive pulmonary disease, who would be considered by the authors of the Quality of Communication Questionnaire to be terminally ill due to chronic disease.^(21,22)^

### Quality of medical communication

Table 2 shows the mean scores for each QoC item, as well as the QoC total score and subscale scores.

**Table 2:** Scores given by patients for each item of the Quality of Communication Questionnaire and the resulting total and subscale scores

Responses to the QoC statement: "When talking with the doctor about important issues like becoming very ill, how good is he/she at":	Mean (SD)	Median (P_25 - 75_)
1. Using words you understand	8.1 (2.1)	8.0 (7.0 - 10.0)
2. Looking you in eye	9.0 (1.3)	10.0 (8.0 - 10.0)
3. Answering all questions about illness	8.1 (2.2)	8.0 (7.0 - 10.0)
4. Listening to what you have to say	8.5 (1.8)	9.0 (7.0 - 10.0)
5. Caring about you as a person	9.1 (1.9)	10.0 (9.0 - 10.0)
6. Giving you their full attention	8.8 (1.6)	10.0 (8.0 - 10.0)
7. Talking about your feelings about getting sicker	1.2 (3.1)	0.0 (0.0 - 0.0)
8. Talking about details if you got sicker	5.1 (4.2)	7.0 (0.0 - 9.0)
9. Talking about what dying might be like	1.3 (3.3)	0.0 (0.0 - 0.0)
10. Talking about how long you have to live	2.1 (4.0)	0.0 (0.0 - 0.0)
11. Involving you in the discussions about your care	1.6 (3.5)	0.0 (0.0 - 0.0)
12. Asking you about important things in life	2.0 (3.7)	0.0 (0.0 - 0.0)
13. Asking about spiritual, religious beliefs	1.7 (3.7)	0.0 (0.0 - 0.0)
Total score	5.1 (1.3)	5.1 (4.3 - 5.8)
Score in the general communication subscale (items 1 to 6)	8.6 (1.3)	8.8 (7.8 - 9.7)
Score in the end-of-life communication subscale (items 7 to 13)	2.1 (1.8)	1.4 (1.0 - 2.9)

QoC - Quality of Communication Questionnaire; SD - standard deviation; P25-75 - 25th and 75th percentiles. *The items in the Quality of Communication Questionnaire are answered on a scale from zero ("worst imagined") to 10 ("best imagined"). There are two alternative response options: "Did not do" (scored as 0 in the database) and "Does not know" (replaced by the median of the participant's scores in the other items).(21,22) All participants answered items 1, 2, 4, and 6; 1 participant answered "Did not do" for item 3; 5 answered "Do not know" for item 5, and 2 of these also answered "Do not know" for item 12; 87 answered "Did not do" for item 7; 33 for item 8; 70 for item 9; 89 for item 10; 86 for item 11; 77 for item 12; and 81 for item 13. No participant gave a score of 0 to the physician on any of the items; all the zeros came from the answer "Did not do".

The mean QoC score among women was 5.1 (SD = 1.2) and among men, 5.1 (SD = 1.4) (t (101) = -0.1, p = 0.91). On the general communication subscale, the mean score for women was 8.7 (SD = 1.2), and for men it was 8.5 (SD = 1.4) (t (101) = -0.76, p = 0.45). In the end-of-life communication subscale, the mean score among women was 2.1 (SD = 1.8), and among men it was 2.2 (SD = 1.9) (t (101) = -0.34, p = 0.73).

**Figure 1:**
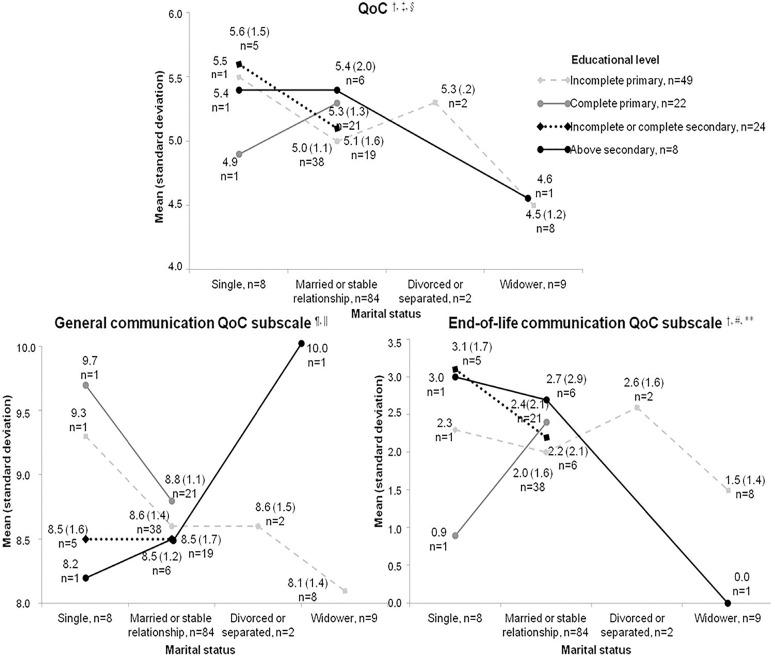
Mean scores of the Quality of Communication Questionnaire* and its subscales among the 103 patients who were or had recently been admitted to the intensive care unit, by marital status and educational level.

Figure 1 shows the QoC total and subscale scores according to educational level and marital status.

QoC - Quality of Communication Questionnaire. *The Quality of Communication Questionnaire items are answered on a scale from 0 (“worst imagined”) to 10 (“best imagined”). There are two response options: “Did not do” (scored as 0) and “Do not know” (replaced by the median of the scores on the other items);(21,22) †all participants answered items 1, 2, 4, and 6; 1 participant answered “Did not do” for item 3; 5 answered “Do not know” for item 5, and 2 of these also answered “Do not know” for item 12; 87 answered “Did not do” for item 7; 33 in 8; 70 in 9; 89 in 10; 86 in 11; 77 in 12; and 81 in 13. No participant gave a score of 0 to the physician in any of the items, so the zeros all came from the replacement of the answer “Did not do”; ‡mean QoC score by marital status: single: 5.5 (SD = 1.2); married/stable relationship: 5.1 (SD = 1.3); divorced/separated: 5.3 (SD = 0.2); widower: 4.5 (SD = 1.1); and by educational level: incomplete primary education: 5.0 (SD = 1.1); complete primary education: 5.3 (SD = 1.3); incomplete or complete secondary education: 5.2 (SD = 1.5); above secondary education: 5.3 (SD = 1.7); §two-way between-group analysis of variance: interaction between marital status and educational level: F (4, 103) = 0.11, p = 0.98; main effect of marital status: F (3, 103) = 0.30, p = 0.82; main effect of educational level: F (3, 103) = 0.29, p = 0.99; ¶mean of the QoC subscale general communication by marital status: single: 8.7 (SD = 1.3); married/in a stable relationship: 8.6 (SD = 1.4); divorced/separated: 8.6 (SD = 1.5); widower: 8.3 (SD = 1.4); and by educational level: incomplete primary education: 8.5 (SD = 1.4); complete primary education: 8.8 (SD = 1.1); incomplete or complete secondary education: 8.5 (SD = 1.6); above secondary education: 8.6 (SD = 1.2); ||two-way between-group analysis of variance groups: interaction between marital status and educational level: F (4, 103) = 0.57, p = 0.68; main effect of marital status: F (3, 103) = 0.24, p = 0.87; main effect of educational level: F (3, 103) = 0.29, p = 0.83; #mean of the QoC subscale end-of-life communication: by marital status: single: 2.7 (SD = 1.5); married/stable relationship: 2.2 (SD = 1.9); divorced/separated: 2.6 (SD = 1.6); widower: 1.3 (SD = 1.4); and by educational level: incomplete primary education: 1.9 (SD = 1.5); complete primary education: 2.3 (SD = 2.0); incomplete or complete secondary education: 2.4 (SD = 2.0); above secondary education: 2.4 (SD = 2.6); **two-way between-group analysis of variance: interaction between marital status and educational level: F (4, 103) = 0.589, p = 0.672; main effect of marital status: F (3, 103) = 0.89, p = 0.45; main effect of educational level: F (3, 103) = 0.29, p = 0.83.

### Perception of patients about communication, their experience, and their needs

**Figure 2:**
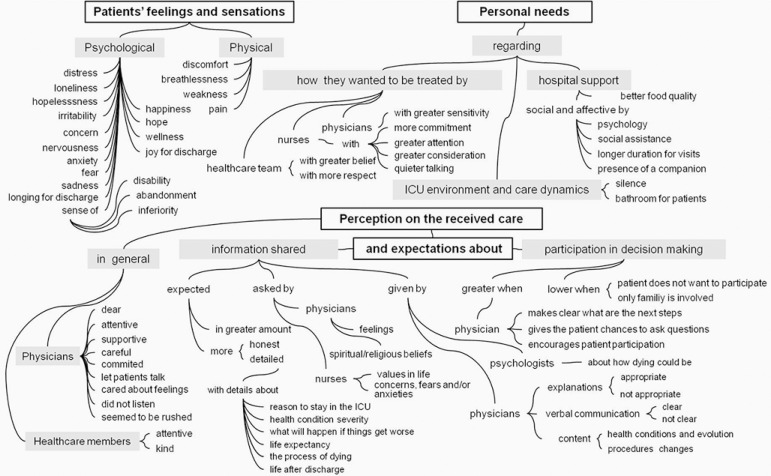
Categories identified from the testimonials of patients who were or had recently been admitted to the intensive care unit and their context units, context subunits, and meaning units.

In the analysis of qualitative data, the categories identified are shown in figure 2, with their context units and subunits.

ICU - intensive care unit.

### Information sharing

Table 3 lists the illustrative statements of patients that fell under the information sharing category, which included their perception of the quality of verbal communication, the explanations/information provided and asked for by physicians and the healthcare team, and the information they would have liked to receive (Table 3).

**Table 3:** Illustrative testimonies about information sharing of patients who were or had been recently admitted to the intensive care unit

1. Information sharing*	Illustrative testimonials
**1.1. Characteristics of the information:**	
**1.1.1. Provided**	
1.1.1.1. Quality of verbal language	
1.1.1.1.1. Clear	"He spoke our language"
1.1.1.1.2. Not clear	"They seem to speak in code"
1.1.1.2. Quality of the information	
1.1.1.2.1. Adequate	"Even without asking, he (the physician) already answers"
1.1.1.2.2. Not adequate	"I don't think they know it well"
	"They say: - 'You have to wait to know'"
1.1.1.3. Content of the information	
1.1.1.3.1. Health condition and progression (physician)	"[...] that if I stop treatment I will have serious lung problems"
1.1.1.3.2. Procedures (physician)	"[...] said that if it can't be solved just (with) a catheter, it will be necessary to perform a bypass surgery"
1.1.1.3.3. Dying process (psychologist)	"Talked about what dying might be like"
**1.1.2. Asked **	
1.1.2.1. Feelings	
1.1.2.1.1. Fear, concern and distress	"Asked if I would be afraid of having to have a new surgery"
1.1.2.2. Beliefs	
1.1.2.2. 1. Religious beliefs and spirituality	"Only one physician asked... (about religious beliefs and spirituality)"
	"Only (asked me) on admission before surgery"
1.1.2.3. Life values	
1.1.2.3. Important things in everyday life	"Only the nurse asked me... (about important things in life)"
**1.1.3. Desired**	
1.1.3.1. Overall	
1.1.3.1.1. In greater quantity and frequency	"(I wanted) more information [...] from the physicians so I didn't need to ask the nurses "
1.1.3.1.2. More honest and detailed	"More honest information... Sometimes, I think there is something more serious"
1.1.3.1.3. Reason for ICU stay	"I don't know the reason I spent so much time in the ICU [...] I don't think so much time is necessary"
1.1.3.1.4. Severity of the problem and possibility of death	"I wanted to know if it was serious or not [...] if I'm going to die or not"
1.1.3.2. Details about	
1.1.3.2.1. What would happen if got sicker	"Wow, how I wanted to know... (details of my condition if I get sicker)"
1.1.3.2.2. Life expectancy	"I know there's no cure, but I wanted at least a number"
1.1.3.2.3. Life after discharge	"If, when I get home, I will be able to climb stairs or [...] will need to adapt the house [...]"
1.1.3.2.4. Dying process	"I truly wanted to know about the suffering before I die, how it will be..."

ICU - intensive care unit..

* The categories are in bold, the context units are in bold italics, the context subunits are in italics, and the meaning units are in normal font.

Some patients participated in decision-making when the physician made clear the following steps of care and gave them the opportunity to ask questions. While some patients reported that the information was passed on only to the family, despite their desire to know more, other patients mentioned that they did not want to participate in this process.

### Perception on the care provided in the intensive care unit

Patient satisfaction with the care received was expressed with comments such as “I had no idea that the hospital was so good, despite being part of the public health system (Unified Health System - SUS)”. The qualities of the physicians were described with terms such as “dear”, “attentive”, “supportive”, “committed”, “careful”, and “cared about feelings”. One patient reported that a physician always asked her if she was well and told her “not to be sad...that it was almost over”. Some patients, however, mentioned that this treatment varied, as some physicians did not listen to them, seeming to be “rushed”. Other healthcare team members were also described as “kind” and “attentive”.

### Patients’ experiences

The experiences reported by the patients often associated physical aspects, such as weakness, pain, breathlessness, and discomfort, with negative emotional aspects, such as fear, concern, distress, anxiety, nervousness, irritability, sadness, sense of disability, hopelessness, sense of abandonment, loneliness, and sense of inferiority. Positive emotional aspects were also reported, such as hope, wellness, happiness, and joy with the discharge from the ICU.

### Patients’ needs

Several needs were reported. One of them was about how patients would like to be treated: with greater physicians’ sensitivity; with more consideration, attention, and commitment by nursing staff; and with more respect and greater belief by the health team. This was illustrated by the following statement: “I’d like them to trust my word when I say I feel pain or some discomfort.”

The need for social and affective support was felt, including more and longer visits and the presence of a companion in the ICU. Another needed support was psychological care for mental health and social work care for work and retirement arrangements. The need for better support services was also mentioned, especially the desire for better quality food.

Several patient needs were related to the ICU environment and dynamics. A bathroom for patients was one of them because, in one patient’s words, it is “uncomfortable to wear a diaper when one can go to the bathroom by themselves [...] it gives a sense of disability.” Another frequently mentioned need was that of silence because, in addition to the noise from ICU devices, certain team members were loud, preventing patients from sleeping and giving them the feeling of not caring about them, as illustrated below:

“If they could speak more quietly... I can’t sleep! The staff talks and laughs very loudly. Sometimes I think the nurses don’t care.”

“I’d like the environment to be quieter, at least at night... I can’t sleep... there’s a lot of loud laughing. And during the day it’s even worse! So I can’t sleep neither at night or during the day. ”

## DISCUSSION

The mean score of QoC subscale 1, related to general communication, was high. Aspects that contributed to this are expressed in patients’ testimonies, such as clear language and attitudes that demonstrated the attention, affection, and commitment of the physicians and the team. These characteristics have also been found in other studies.^(1,3,10,11,17)^

The mean score on QoC subscale 2, end-of-life communication, was low and decreased the total QoC mean score. This occurred because only seven of the 103 patients were in the end-of-life, but also among them it was extremely frequent the response “Did not do” in the items of this subscale.. As the answer “Did not do” is represented by a score of 0, this raises great concern about the validity of these items because a score of 0 for the answer “Did not do” differs greatly from a score of 0 given by the patient for communication about a given item. Therefore, only QoC subscale 1, general communication, seems to be more appropriate to the patients studied.

There was no difference in the QoC total or subscales mean scores by sex, educational level or marital status. These results in regard to sex are similar to those of other studies.^(19,26-29)^ Regarding the educational level, some studies have similar results,^(19,26,30)^ while others suggest that a higher level leads to less difficulty and greater satisfaction with communication.^(28,29,31,32)^ Regarding marital status, in some studies, unmarried patients had lower scores in regard to communication perception or quality.^(29,30)^

Regarding shared information, while some patients were satisfied with the information received about procedures and therapeutic plans, others considered it insufficient, similar to what was found by Santiago de Castro and Vargas Rosero in Colombia.^(3)^

Some patients in the present study also reported the appropriate provision of information about the disease and treatment and questions asked about their feelings, concerns, important things in their lives, and their spiritual and religious beliefs.

Several studies suggest that the healthcare team should provide frequent information about people, time, and the surrounding environment to patients in the ICU; talk about everyday life to help them stay in touch with reality and strengthen their desire to return to normal life and increase their hope;^(1,2,4,10,11,18)^ and address spirituality, which is a source of hope and security.^(3,4)^

Even among patients who praised the team, aspects that could be improved were noted. These included less hurry and more time available to answer questions about the clinical condition, treatment, and prognosis, with more information being provided more frequently and with more detail and honesty.

A study in Sweden also found that some patients admitted to the ICU found certain team members “more interested in solving their own work schedule than in taking care of the patients,” and, although they considered the information provided clear, they did not find it sufficient because they wanted to know more about the reasons for admission/stay in the ICU and the exams performed.^(11)^ Other studies also indicate insufficient provision of information.^(1-5,7-9,11,12,14,17)^ In one of them, some patients reported not understanding the information provided, which caused them anxiety and distress.^(4)^

Regarding the patients’ participation in decision-making, while some physicians encouraged them to speak, express their doubts, and participate in this process, making clear the next care steps, other physicians talked only with the family, regardless of the patient’s desire. Some patients, however, truly did not want to participate in this process.

Fewer than half of the patients in a study in Estonia^(17)^ and in Jordan^(1)^ and one-third of patients in the study in Sweden^(11)^ felt that they had no control over decisions about their care or that their opinion was important. This could be because, when admitted to ICUs, patients believe that important clinical decisions should be the responsibility of the professionals who care for their health.^(1,5,17)^ Based on their study, Wåhlin et al. argue that although important decisions on technical issues are usually delegated to health professionals, making decisions about aspects of daily care, such as personal hygiene schedule, can be valuable for patients, so their involvement should be encouraged.^(4)^

Regarding what the patients felt, the findings of this study agree with other studies with patients admitted to the ICU, including pain and discomfort,^(1-6,8-15,17-19)^ loneliness,^(3,5,9-11,13,15-18)^ anxiety,^(2-4,6,8-11,13,14,17-19)^ and fear.^(1,3,5,8,9,13,15-19)^ Alasad et al. raised concerns about the possibility of insufficient pain control in ICU patients.^(1)^ Faria et al. highlighted that pain management was the most cited measure in studies they reviewed.^(9)^

Positive feelings, such as well-being, hope, happiness for having been or soon being discharged from the ICU, were also found in the Colombian study.^(3)^

Several needs were mentioned by the patients, such as greater commitment, sensitivity, consideration, and attention by some team members and greater respect and belief when they reported feeling pain or discomfort.

The disbelief by some members of the healthcare team when patients reported pain or difficulty breathing was also pointed out by patients admitted to the ICU in the study by Wåhlin et al., in which it was also mentioned that some professionals showed involvement, encouragement, and concern for their comfort.^(11)^

Social and affective support were another needs reported in this study. This has also been expressed in several studies that emphasize the importance of more and longer visits and the presence of a companion.^(1,3,5,7-11,13,15-18)^ These are considered protective factors for patients because they increase their connection with the “real” world in a scenario in which one of the most frequently cited feelings is the disconnection with reality; they generate a greater sense of security, facilitate the reframing of their life, provide inner strength, help to strengthen the spirit,^(1,3-6,8,10,11,15,17,18)^ facilitate patients’ understanding of the information provided by the team, and help the team to better understand patients’ history, needs, and perspectives.^(17)^

Other need was for support by members of the multidisciplinary team, among them psychologists for the preservation or treatment of mental health; social workers to assist in making work- and retirement-related arrangements; and nutrition services to provide better quality food. Carrese et al. performed direct observations in two American ICUs and noted, among other factors, the need for better food quality.^(12)^

The need for a bathroom for patients in the ICU was mentioned, especially among those who could use it by themselves. Due to the lack of a bathroom, the patients were forced to wear diapers, making them feel uncomfortable and disabled. Other studies have also found discomfort among patients due to the lack of bathrooms, not only because they have to relieve themselves in diapers or in previously unknown devices (such as male and female urinals) but also because they feel their privacy is violated when their private parts are exposed to unknown people. In addition, baths in the ICU often have to be performed in the bed, with even greater body exposure, sometimes to patients in neighboring beds, which worsens their discomfort.^(1-3,5,8-10,12-15,17)^ Aro et al. suggest that patients should at least be separated from others with screens during these procedures in order to provide them some privacy.^(17)^

The need for silence was highlighted because, while noise from ICU equipment was inevitable, other sources of noise could be avoided, especially loud conversations and laughter from the team members, which, in addition to preventing patients from sleeping and resting, made them feel disrespected. Noise has been noted in other studies, coming from alarms, telephones, monitoring devices, and conversations between healthcare team members.^(1,3,5,8,10-15,17,18)^ In the study with 45 patients admitted to an ICU in Colombia, noise was reported by 80% of them.^(10)^ In another study, the ICU environment was described as “a war zone, only with patients”.^(5)^

Although not mentioned in the present study, other aspects that hinder patient rest include lighting from lights at night^(11)^ and the room temperature of the ICU. According to Carrese et al., the adequacy of the physical environment is critical for ethical, dignified, and respectful treatment of patients.^(12)^

This study shows that the spontaneous testimonies of patients can complement their quantitative evaluations of the quality of medical communication, which received good scores in regard to the general communication. Nevertheless, the testimonies indicated that there are important aspects to be improved to promote care quality and highlighted the patients’ needs regarding communication and their care.

### Limitations

The limitations of this study were the convenience sampling of patients and the fact that not all of them made spontaneous comments regarding the QoC items. However, because the statements were spontaneously made, they added valuable information, enabling greater understanding of the process of communication between the physicians and other healthcare team members and patients in the ICU.

## CONCLUSION

The general communication with the physician, assessed by subscale 1 of the Quality of Communication questionnaire, was good. The end-of-life communication subscale was not considered valid because most patients answered “Did not do” (given score 0) for its items, which did not allow the evaluation of an assigned score.

Clarity in language, opportunities to ask questions and clarify doubts, information sharing in a detailed and honest manner, and addressing emotional and spiritual issues were aspects of medical communication valued by patients.

The care received in the intensive care units exceeded some patients’ expectations, but others did not feel heard or thought that some team members seemed to be rushed or did not care for them. Some patients mentioned the need for greater commitment, attention, sensitivity, respect, and belief in what they said and felt by certain team members.

Other needs included more and longer visits, the presence of a companion, support by professionals from the multidisciplinary team, better food quality, silence for resting and as a form of respect, and bathrooms that patients could use for greater comfort and privacy.

We hope that the knowledge generated here can serve for the improvement of patient care in the ICU, which should be focused on interpersonal relationships and respect for human dignity.
